# Association of a functional microsatellite within intron 1 of the *BMP5 *gene with susceptibility to osteoarthritis

**DOI:** 10.1186/1471-2350-10-141

**Published:** 2009-12-19

**Authors:** James M Wilkins, Lorraine Southam, Zehra Mustafa, Kay Chapman, John Loughlin

**Affiliations:** 1 University of Oxford, Institute of Musculoskeletal Sciences, Botnar Research Centre, Nuffield Orthopaedic Centre, Oxford, OX3 7LD, UK; 2 Newcastle University, Institute of Cellular Medicine, Newcastle-upon-Tyne, NE2 4HH, UK

## Abstract

**Background:**

In a previous study carried out by our group, the genotyping of 36 microsatellite markers from within a narrow interval of chromosome 6p12.3-q13 generated evidence for linkage and for association to female hip osteoarthritis (OA), with the most compelling association found for a marker within intron 1 of the bone morphogenetic protein 5 gene (*BMP5*). In this study, we aimed to further categorize the association of variants within intron 1 of *BMP5 *with OA through an expanded genetic association study of the intron and subsequent functional analysis of associated polymorphisms.

**Methods:**

We genotyped 18 common polymorphisms including 8 microsatellites and 9 single nucleotide polymorphisms (SNPs) and 1 insertion/deletion (INDEL) from within highly conserved regions between human and mouse within intron 1 of *BMP5*. These markers were then tested for association to OA by a two-stage approach in which the polymorphisms were initially genotyped in a case-control cohort comprising 361 individuals with associated polymorphisms (*P *≤ 0.05) then genotyped in a second case-control cohort comprising 1185 individuals.

**Results:**

Two *BMP5 *intron 1 polymorphisms demonstrated association in the combined case-control cohort of 1546 individuals (765 cases and 781 controls): microsatellite D6S1276 (*P *= 0.018) and SNP rs921126 (*P *= 0.013). Functional analyses in osteoblastic, chondrocytic, and adipocytic cell lines indicated that allelic variants of D6S1276 have significant effects on the transcriptional activity of the *BMP5 *promoter *in vitro*.

**Conclusion:**

Variability in gene expression of *BMP5 *may be an important contributor to OA genetic susceptibility.

## Background

Osteoarthritis (OA, MIM 165720) is the most common musculoskeletal disorder in developed countries [1]. Pathologically, OA is characterized by the focal degeneration of the smooth articular cartilage in any of the synovial joints of the body with the hand, spine, knee, and hip the most frequently affected joint sites [2]. In addition to cartilage damage, OA is associated with a variety of changes in other joint tissues, such as new bone formation at the joint margins (osteophytes), subchondral bone sclerosis, and joint capsule thickening, with effects also seen in the ligaments and peri-articular muscles and tendons [2,3].

Although the underlying molecular mechanisms for disease initiation and progression are still not yet fully understood, epidemiological and population-based cohort studies have evidenced a significant genetic component to OA susceptibility [4]. Genome-wide linkage scans and large-scale association studies have had some success in unravelling the genetic architecture underlying OA with the identification of a number of susceptibility genes [5,6]. These efforts and subsequent replication studies, however, have done more to highlight the complex nature of OA genetic susceptibility whereby sex-specific, joint-specific, and population-specific genetic predispositions have been shown to exist [5,7].

In a previous study carried out by our group, the genotyping of 36 microsatellite markers from within a narrow interval of chromosome 6p12.3-q13 generated evidence for linkage (*P *= 0.000001) and for association (*P *= 0.007) to female hip OA, with the most compelling association observed for marker D6S1276 located within intron 1 of the bone morphogenetic protein 5 gene (*BMP5*) (MIM, 112265) [8]. BMP5 is a member of the TGF-β superfamily of secreted proteins whose family members are involved in synovial joint development and joint tissue homeostasis [9]. Polymorphisms located within the transcribed region of *BMP5 *and within its proximal promoter had previously been excluded for association with OA [10], so our association to intron 1 of *BMP5 *was unlikely to be explicated by linkage disequilibrium (LD) between D6S1276 and polymorphism/s within the coding region or promoter of the gene. There is increasing evidence, however, that polymorphisms in regulatory elements involved in gene transcription play an important role in conferring susceptibility to complex disease traits [11]. Thus, it seems plausible that the OA susceptibility mapped to intron 1 of *BMP5 *may be due to polymorphisms in *cis*-regulatory elements that act by quantitatively altering gene expression as opposed to amino acid substitutions that qualitatively alter the structure of the encoded protein.

In the present study, we aimed to further categorize the association of variants within intron 1 of *BMP5 *with female hip OA through an expanded genetic association study of the intron. We increased the number of polymorphic markers within intron 1 to include additional microsatellite markers as well as single nucleotide polymorphisms (SNPs) and insertion/deletions (INDELs) from within areas of high sequence conservation between human and mouse, as inter-species comparisons have generally proven successful in identifying functional non-coding elements in the human genome [12]. Through this analysis, we identified a SNP and a functional microsatellite associated with OA and show that allelic variants of the microsatellite are responsible for altered transcriptional activity of the *BMP5 *promoter, which implies that polymorphism in *cis*-regulation of *BMP5 *is involved in OA susceptibility.

## Methods

### Subjects

Female hip OA cases were ascertained through the Nuffield Orthopaedic Centre in Oxford, UK based on inclusion criteria of symptoms of primary OA sufficiently severe to require total hip replacement (THR). All cases had pain with rest and night symptoms for more than 6 months duration. The radiological stage of the disease was Kellgren-Lawrence grade 2 or more in all cases with over 90% of cases being grade 3 or 4. Inflammatory arthritis (rheumatoid, polyarthritic, or autoimmune disease) was excluded, as was post-traumatic or post-septic arthritis. No cases suggestive of skeletal dysplasia or developmental dysplasia were included. The average age of the cases at THR was 64 years with an age range of 58-84 years. For the case-control investigation, a two-stage association analysis was performed.

For case-control cohort 1 (CCC1), 146 female hip OA cases that were the probands from the female families used in our original linkage analysis as well as the 215 age-matched female controls from that study were analyzed [8]. For case-control cohort 2 (CCC2), an additional 619 female hip OA cases and 566 female controls were analyzed. The controls of CCC2 had an unknown OA status and were not age-matched with the cases. All together, 765 unrelated female hip OA cases and 781 controls were recruited for this study. All cases and all controls were UK Caucasians. Ethical approval for this study was obtained from the Central Oxford Research Ethics Committee.

### Case-control association analysis

For the case-control association analysis, genomic DNA was extracted from whole blood samples using standard methods. Microsatellites were PCR amplified and hybridized with fluorescently-labelled primers. Primer sequences and PCR conditions are available from the authors. The fluorescently-labelled products were separated and visualized using an Applied Biosystems 3100 Genetic Analyzer (Applied Biosystems). Microsatellite genotypes were determined using GeneMapper 3.5 software (Applied Biosystems). The SNPs and INDEL were genotyped by PCR-restriction fragment length polymorphism (RFLP) analysis with the digestion products electrophoresed through 3% agarose gels, stained with ethidium bromide, and scored following UV visualization. For each RFLP analysis, approximately 96 individuals were sequenced to ensure correct genotyping in the RFLP assay. Primer sequences, restriction enzymes, and PCR conditions are available from the authors.

The CLUMP program was used to test each microsatellite marker for association with female hip OA [13]. Significance was assessed for each marker by performing 1,000 simulations to generate tables with the same marginal totals as the original data. Empirical *P *values were obtained by counting the number of times the χ^2 ^value of the real data was achieved by the simulated tables. CLUMP calculates 4 statistics: T1-T4. T1 is a standard contingency table χ^2^, T2 is a χ^2 ^calculated after collapsing together alleles with a small expected value (< 0.05), T3 is the largest 2 × 2 χ^2 ^in which each allele is compared in turn with the rest grouped together, and T4 is the largest 2 × 2 χ^2 ^in which alleles demonstrating a frequency difference between cases and controls are clumped together and compared with the remainder. For SNPs and INDELs, allele distributions in cases and controls were compared using standard χ^2 ^analysis-of-contingency tables. Hardy-Weinberg equilibrium for the distribution of genotypes was confirmed for these data using standard χ^2 ^analysis-of-contingency tables. Odds ratios (ORs) were calculated with 95% confidence intervals (CIs). Frequencies of multilocus haplotypes were estimated with EH-PLUS [14], and these haplotypes were tested for association with female hip OA using the CLUMPHAP program [15], which is an extension of the basic methodology of the CLUMP program. CLUMPHAP works by collapsing similar haplotypes together into two groups to obtain the largest χ^2 ^statistic from which an empirical *P*-value is obtained by permutation.

### Construction of luciferase reporter plasmids

To generate constructs for use in luciferase reporter gene assays, a 1,311 bp fragment of genomic DNA corresponding to the *BMP5 *proximal promoter (-1783 to -472 with respect to the translation start site of *BMP5*) was PCR amplified using gene-specific primers containing either a *Kpn*I (forward primer) or a *Nhe*I (reverse primer) restriction site at the 5' end of the primer. The source of this genomic DNA was one of the individuals used in our study. Primer sequences can be found in Additional file 1, Table S1. After PCR amplification and double digestion with *Kpn*I and *Nhe*I restriction enzymes, the *Kpn*I-*Nhe*I promoter fragment was purified using the QIAquick PCR purification kit (Qiagen). This fragment was then cloned into the *Kpn*I/*Nhe*I sites of the pGL3-Basic luciferase reporter vector (Promega) using the Clonables Ligation/Transformation kit (Novagen) to create the Promoter-Luciferase construct (Additional file 1, Figure S1). Plasmid DNA used for transfections was isolated from positive clones using the GenElute HP Plasmid MidiPrep Kit (Sigma) and sequenced to ensure that the plasmid contained the correct nucleotide sequence.

PCR primers encompassing intronic polymorphisms D6S1276, rs921126, and rs17734678 were designed with the forward primer containing a *Nhe*I restriction site and the reverse primer containing a *Hind*III restriction site at the 5' end of the primers. Primer sequences can be found in Additional file 1, Table S1. Genomic DNA from a case or a control containing the allele of interest was PCR amplified, double digested with restriction enzymes *Nhe*I and *Hind*III, purified, and cloned into the *Kpn*I/*Hind*III sites of the Promoter-Luciferase construct (Additional file 1, Figure S1). These constructs were isolated and sequenced to ensure the correct nucleotide sequence had been cloned. Site-directed mutations of the DS1276 clones F-I and F-II were performed with the QuikChange II Site-Directed Mutagenesis kit (Stratagene) following the manufacturer's instructions. Primers used for the mutagenesis reaction were designed with the QuickChange Primer Design Program http://www.stratagene.com/qcprimerdesign/. Primer sequences can be found in Additional file 1, Table S2. Mutated clones were sequenced to verify the appropriate mutations had been made.

### Cell culture and luciferase assay

All cells were maintained at 37°C in a humidified 5% CO_2 _incubator. MG63 cells (human osteosarcoma) and CH8 cells (human articular chondrocytes) [16] were cultured in Dulbecco's modified Eagle medium (DMEM) (Cambrex) supplemented with 10% fetal calf serum (FCS). CH8 cells were maintained on type-I collagen coated culture dishes (BD Biosciences). SW872 cells (human liposarcoma) were cultured in DMEM/F-12 (3:1) (Cambrex) with 5% FCS. Cells were seeded at a density of approximately 3 × 10^5 ^cells per well of a 6-well cluster plate (Appleton Woods) 24 hours before transfection. CH8 cells were plated in type-I collagen coated 6-well plates (BD Biosciences). Using the GeneJuice transfection reagent (Novagen), cells were transfected with 1 μg of the luciferase reporter construct and 1 μg of pSV-β-galactosidase control vector (Promega) as an internal control to normalize for transfection efficiency. Twenty-four hours after transfection, the transfection mixture was removed and replaced with complete growth medium. After an additional 24 hours, the cells were washed once with phosphate-buffered saline (PBS) and lysed with 250 μl of 1X Reporter Lysis Buffer (Promega) per well.

Cellular extracts were prepared and 20 μl of cell lysate was assayed for luciferase activity with the Luciferase Assay System (Promega) using a GloMax 20/20 Luminometer (Promega). β-galactosidase activity was measured with a spectrophotometric assay using 10 μl of the same cell lysate and 150 μl of 2-nitrophenyl β-D-galactopyranoside (ONPG) solution (2.9 mM ONPG, 1 mM MgCl_2_, 14.1 mM β-mercaptoethanol, 82 mM Na_2_HPO_4_·2H_2_O, and 18 mM NaH_2_PO_4_·2H_2_O) as substrate. Absorbencies were measured at 420 nm and then compared to a standard curve of know quantities of β-galactosidase. Transfections were performed in triplicate on at least three separate occasions. Relative luciferase activity for each transfection was determined by taking the ratio of luciferase activity to β-galactosidase activity, and these data were subsequently normalized by the mean of the relative luciferase activities of the Promoter-Luciferase construct for each cell line. Statistical comparisons were performed with the two-tailed Student's *t*-test. The Bonferroni correction was applied for multiple testing.

## Results

### Association Analysis

Seventeen polymorphic markers within intron 1 of *BMP5 *(9 SNPs, 1 INDEL, and 7 microsatellites) were identified for use in the case-control association analysis (Additional file 1, Table S3). We focused our search for SNPs and INDELs on areas of DNA sequence homology between human and mouse within intron 1 of *BMP5 *by comparing the 54 kb genomic region encompassing intron 1 of *BMP5 *with the corresponding mouse sequence using the VISTA Genome Browser http://pipeline.lbl.gov/[17] with search criteria of at least 70% sequence identity over at least 100 base pairs in length. Through this search, we identified 28 conserved non-coding sequences (Additional file 1, Table S4), and within these conserved sequences, 9 common SNPs and 1 common INDEL (minor allele frequency > 5%) were identified using the UCSC Human Genome Browser http://genome.ucsc.edu/[18] and direct DNA sequencing. To identify microsatellite markers within intron 1 of *BMP5 *for use in the association analysis, we searched the UCSC Human Genome Browser May 2004 human reference sequence, NCBI build 35 for simple tandem repeats consisting of di-, tri-, tetra-, or pentanucleotide repeats. The polymorphic nature of these microsatellites was confirmed by genotyping at least 32 Caucasian chromosomes, and microsatellites having a heterozygosity value of 30% or greater were subsequently included in the association study.

The 17 polymorphic markers along with the microsatellite D6S1276 were initially genotyped in case-control cohort 1 (CCC1). Two pairs of SNPs were found to demonstrate complete LD (pair-wise *r*^2 ^= 1.0): rs17734678 with rs921126 and rs3798818 with rs9382564 (Additional file 1, Table S5), and consequently only one of the two SNPs from each pair was studied further in the association analysis. Of the 18 polymorphisms genotyped, 5 demonstrated significant association (*P *≤ 0.05) with female hip OA in CCC1: microsatellite D6S1276 and SNPs rs921126, rs1470527, rs3798821, and rs9382564 (Table 1). These 5 polymorphisms were then genotyped in case-control cohort 2 (CCC2), with none of these polymorphisms demonstrating association with CCC2 alone. When CCC1 and CCC2 were combined, however, D6S1276 (*P *= 0.018, CLUMP T4) and rs921126 (*P *= 0.013; OR 1.23, 95% CI 1.04-1.47) demonstrated association with the combined cohort, and rs1470527 showed a trend toward association (*P *= 0.078) (Table 1). For the analysis of D6S1276, the T4 statistic produced the strongest evidence of association, which was achieved by clumping together alleles B, C, E, F, G, H, and K (Table 2). Table 2 presents the allele counts and frequencies (%) for D6S1276 and rs921126 in CCC1, CCC2, and the combined case-control cohort.

**Table 1:** Association analysis of polymorphisms within intron 1 of *BMP5 *in CCC1, CCC2, and the combined data.

Marker	CCC1					CCC2					Combined Cohort				
	
	Microsatellite *P *by CLUMP				SNP *P *by χ^2^	Microsatellite *P *by CLUMP				SNP *P *by χ^2^	Microsatellite *P *by CLUMP				SNP *P *by χ^2^
						
	T1	T2	T3	T4		T1	T2	T3	T4		T1	T2	T3	T4	
rs921126					**0.022**					0.16					**0.013**
rs1470527					**0.044**					0.41					0.078
D6S1276	**0.007**	**0.008**	**0.03**	0.06		0.25	0.17	0.20	0.14		**0.041**	**0.025**	**0.040**	**0.018**	
rs3798821					**0.004**					0.74					0.23
rs9382564					**0.025**					0.71					0.14

*P *values ≤ 0.05 are highlighted in **bold**.

**Table 2:** Allele counts and allele frequencies for polymorphisms D6S1276 and rs921126 in CCC1, CCC2, and the combined case-control cohort

Marker	Allele	Repeat	Allele Counts (%)						
			
			CCC1		CCC2		Combined Cohort		Total Chromosomes
				
			Cases	Controls	Cases	Controls	Cases	Controls	
D6S1276	A	(TCTA)_3_	0 (0.0)	1 (0.2)	0 (0.0)	0 (0.0)	0 (0.0)	1 (0.1)	1 (0.0)
	B	(TCTA)_5_	0 (0.0)	0 (0.0)	1 (0.1)	0 (0.0)	1 (0.1)	0 (0.0)	1 (0.0)
	C	(TCTA)_6_	1 (0.4)	0 (0.0)	1 (0.1)	2 (0.2)	2 (0.1)	2 (0.1)	4 (0.1)
	D	(TCTA)_7_	2 (0.8)	15 (3.5)	34 (2.8)	45 (4.0)	36 (2.5)	60 (3.9)	96 (3.2)
	E	(TCTA)_8_	66 (26.0)	73 (17.2)	264 (21.9)	238 (21.4)	330 (22.6)	311 (20.2)	641 (21.4)
	F	(TCTA)_9_	41 (16.1)	83 (19.6)	241 (20.0)	200 (18.0)	282 (19.3)	283 (18.4)	565 (18.9)
	G	(TCTA)_10_	106 (41.7)	189 (44.6)	497 (40.3)	450 (40.5)	603 (41.4)	639 (41.6)	1242 (41.5)
	H	(TCTA)_11_	38 (15.0)	56 (13.2)	156 (13.0)	157 (14.1)	194 (13.3)	213 (13.9)	407 (13.6)
	I	(TCTA)_12_	0 (0.0)	7 (1.7)	7 (0.6)	17 (1.5)	7 (0.5)	24 (1.6)	31 (1.0)
	J	(TCTA)_13_	0 (0.0)	0 (0.0)	2 (0.2)	3 (0.3)	2 (0.1)	3 (0.2)	5 (0.2)
	K	(TCTA)_15_	0 (0.0)	0 (0.0)	1 (0.1)	0 (0.0)	1 (0.1)	0 (0.0)	1 (0.0)

rs921126	A	----	81 (27.9)	87 (20.2)	333 (26.9)	272 (24.3)	414 (27.1)	359 (23.1)	773 (25.1)
	G	----	209 (72.1)	343 (79.8)	907 (73.1)	846 (75.7)	1116 (72.9)	1189 (76.9)	2305 (74.9)

### Haplotype analysis of D6S1276

**Figure 1:**
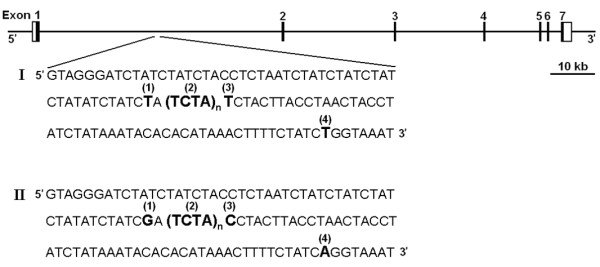
**Genomic structure of the *BMP5 *gene**. The exons are represented by boxes with black fields depicting coding regions and white fields representing the untranslated regions. Solid lines represent introns and intergenic regions. The positions of rs9475431 (1), D6S1276 (2), rs9475430 (3), and rs9475429 (4) are shown with flanking sequence within intron 1 of *BMP5 *along with the two common haplotypes comprised of rs9475431, rs9475430, rs9475429: haplotype I (TTT) and haplotype II (GCA).

D6S1276 is a complex tetranucleotide repeat microsatellite with a backbone repeat element TCTA and three SNPs in close proximity to the microsatellite: rs9475431, rs9475430, and rs9475429 (Figure 1). These three SNPs are in complete LD with each other (pair-wise *r*^2 ^= 1.0) and in very strong LD with rs1470527 (pair-wise *r*^2 ^= 0.96), one of the SNPs used in our association analysis (Additional file 1, Table S5). The haplotype structure of rs9475431, rs9475430, and rs9475429 was analyzed and two common haplotypes were identified, which are depicted as haplotype I and haplotype II in Figure 1. Because of the tight linkage between rs1470527 and the three SNPs proximal to D6S1276, haplotype I was tagged by the T allele of rs1470527 and haplotype II was tagged by the C allele of rs1470527. Haplotypes were then constructed between rs1470527, rs9475431, rs9475430, rs9475429, and D6S1276 in the combined cases and controls of the cohort used for our association analysis. Through this analysis, 17 haplotypes were identified, although only 9 had a frequency ≥ 1% (Additional file 1, Table S6). These haplotypes were tested for association with female hip OA using the CLUMPHAP program. From the analysis of the *BMP5 *haplotype data, an empirical P-value of 0.24 was obtained after 9999 permutations.

### Analysis of allelic variation of D6S1276 on transcriptional regulation of *BMP5*

**Figure 2:**
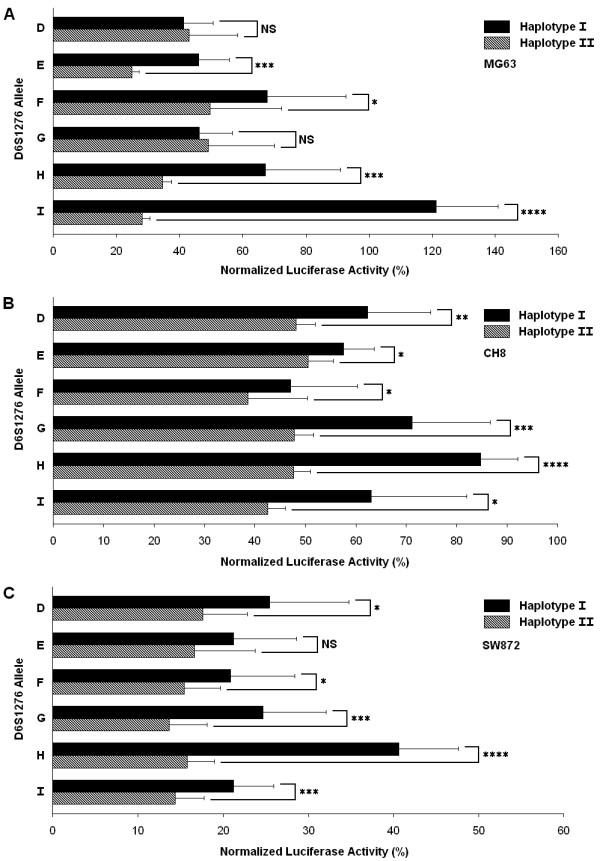
**Effect of microsatellite D6S1276 polymorphism on *BMP5 *promoter activity**. Luciferase reporter assays of the D6S1276 constructs were performed in MG63 (A), CH8 (B), and SW872 cells (C). Transcriptional activities are given as a percentage of the activity of the Promoter-Luciferase construct for each cell line. Data shown are the mean ± SD of at least 3 experiments done in triplicate. Black bars indicate results for haplotype I (rs9475431 T, rs9475430 T, and rs9475429 T). Checked bars indicate results for haplotype II (rs9475431 G, rs9475430 C, and rs9475429 A). *P < 0.05, ** *P *< 0.01, *** *P *< 0.005, **** *P *< 1.0 × 10^-6^, NS is not significant (Student's t-test).

To determine if the D6S1276 alleles had any effect on the transcriptional activity of *BMP5*, luciferase reporter gene assays were performed. Alleles with a frequency ≥ 1% (D, E, F, G, H, I) from Table 2 were chosen, and for these alleles, the two haplotypic forms were analyzed. A 1.311 kb stretch of *BMP5 *that had previously been shown to have promoter activity [19] was first subcloned into the pGL3-Basic vector upstream of the luciferase gene to create the *BMP5 *Promoter-Luciferase construct. This construct was found to have significant promoter activity compared to the pGL3-Basic vector in all of the cell lines analyzed (Additional file 1, Figure S2). The various haplotypes of D6S1276 and SNPs rs9475431, rs9475430, and rs9475429 were then subcloned into the *BMP5 *Promoter-Luciferase construct and transiently transfected into MG63 (human osteosarcoma), CH8 (human articular chondrocytes), and SW872 (human liposarcoma) cells (Figure 2). This analysis demonstrated that not only is D6S1276 a functional microsatellite, but also that there is a significant amount of inter-allelic, intra-allelic, and cell-specific difference in the ability of these alleles to modify transcriptional activity of the *BMP5 *promoter.

A variety of effects on transcriptional activity mediated by the various D6S1276 haplotypes was observed (Figure 2). A number of consistencies across the cell lines were identified with haplotype II-I ((TCTA)_12_) showing one of the lower luciferase activities in all of the cell lines and haplotype I-H ((TCTA)_11_) showing one of the higher luciferase activities in all of the cell lines. There were a number of instances, however, where the transcriptional activity of a particular allele was specific to a specific cell-type. For example, haplotype I of the I allele ((TCTA)_12_) showed a 20% enhancement of luciferase activity relative to the promoter-only vector in MG63 cells but a 40% repression in CH8 cells and an 80% repression in SW872 cells. Additionally, some alleles of D6S1276 demonstrated a significant difference in luciferase activity between haplotype I and haplotype II in some cell-lines but not in others. For example, haplotypes I and II of D6S1276 alleles D ((TCTA)_7_) and G ((TCTA)_10_) showed significant differences in luciferase activity (*P *≤ 0.05) in CH8 and SW872 cells but not in MG63 cells, while haplotypes I and II of D6S1276 allele E ((TCTA)_8_) showed significant differences in luciferase activity in MG63 and CH8 cells but not in SW872 cells. Moreover, the D6S1276 constructs seemed to mediate an increased repression of transcription form the *BMP5 *promoter in SW872 cells relative to the other cell lines (Figure 2), although the *BMP5 *Promoter-Luciferase construct showed the strongest induction of luciferase activity in this cell line (Additional file 1, Figure S2).

**Figure 3:**
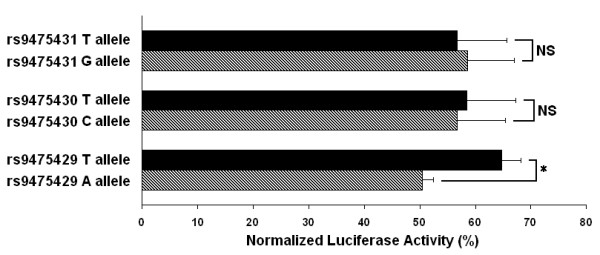
**SNP comparison of rs9475431, rs9475430, and rs9475429 on *BMP5 *promoter activity**. Luciferase reporter assays comparing the mean transcriptional activities of haplotypes containing the T allele of rs9475431 and haplotypes containing the G allele, haplotypes containing the T allele of rs9475430 and haplotypes containing the C allele, and haplotypes containing the T allele of rs9475429 and haplotypes containing the A allele were performed in MG63 cells. Data shown are the mean ± SD. * *P *= 2.4 × 10^-3^. NS is not significant.

This study also allowed for investigation of differences in luciferase activity between haplotypes I and II while holding the length of the allele constant. Figure 2 shows that there is a consistent trend for significantly repressed luciferase activity from constructs carrying haplotype II relative to haplotype I for each D6S1276 allele with as much as a 4-fold repression for haplotype II relative to haplotype I as seen for the I allele ((TCTA)_12_) in MG63 cells (P = 4.6 × 10^-7^). To discern which SNP/s were responsible for the difference in transcriptional activity between haplotype I and II, each of the three SNPs were systematically mutated with site-directed mutagenesis to create all possible haplotypic combinations of rs9475431, rs9475430, and rs9475429 while holding the number of TCTA repeats constant at 9 (F allele of D6S1276). The mutated vectors were then transiently transfected into MG63, CH8, and SW872 cells and assayed for luciferase activity (Additional file 1, Figure S3). To determine the functional contribution of each of the three SNPs, the mean luciferase activities of the haplotypes carrying either allele for each SNP were compared. The mean luciferase activities of haplotypes containing the T allele of rs9475429 were 1.3-fold higher than the activities of haplotypes containing the A allele in MG63 cells (P = 2.4 × 10^-3^) (Figure 3). There was no statistically significant difference, however, in the luciferase activities for haplotypes carrying the T allele or the C allele of rs9475430 or for haplotypes carrying the T allele or the G allele of rs9475431. Similar results were obtained in CH8 cells and in SW872 cells (Additional file 1, Figure S4).

### Functional analysis of rs921126 and rs17734678

To investigate whether the associated SNPs rs921126 and rs17734678 generated any allelic differences in the transcriptional regulation of *BMP5*, fragments of genomic DNA containing the alleles of these SNPs were subcloned into the *BMP5 *Promoter-Luciferase construct and transfected in parallel into MG63, CH8, and SW872 cells. No allelic difference in luciferase activity for rs921126 or rs17734678 was observed in these three cell lines (data not shown). LD mapping of CEPH data using Haploview [20] and the Human HapMap release 21 revealed that these SNPs lie in an LD block of approximately 64 kb, and that within this interval there are at least 28 other SNPs with a pair-wise *r*^2 ^≥ 0.5.

## Discussion

Based on a previous study demonstrating association of a microsatellite marker within intron 1 of *BMP5 *with female hip OA [8], we performed an expanded genetic study of the intron. Association of microsatellite D6S1276 with female hip OA was confirmed and the results described here demonstrated significant variability in the ability of different D6S1276 alleles to modulate the transcriptional activity of the *BMP5 *promoter *in vitro*. Association with female hip OA was also observed for two SNPs showing perfect LD: rs921126 and rs17734678. No significant allelic effect on gene transcription was discovered for either SNP, however, which suggests that these SNPs are not the causal variants but are marking an LD block within which the causal variant/s is likely to reside.

For this study, the CLUMP program [13] was used to compare microsatellite allele frequencies between OA cases and controls. For association analyses, it is recommended that either test statistic T1 or T4 is used as these are reported to be the most powerful and least likely to lose information about differences between cases and controls. For our analysis of D6S1276, the T4 statistic produced the strongest evidence of association, which suggests that OA susceptibility is mediated by multiple D6S1276 alleles as T4 is calculated to maximize the χ^2 ^value by comparing a variable number of alleles against the rest in all possible 2 × 2 tables. The putative involvement of multiple D6S1276 alleles in OA susceptibility is further strengthened by our *in vitro *data that revealed allele-specific control of *cis-*regulation. Additionally, association analysis of rs9475429 (via rs1470527) and haplotype analysis of D6S1276 and rs9475429 showed no significant association, suggesting that although rs9475429 is functional, OA susceptibility mediated by D6S1276 has more to do with the particular size of the allele than its haplotypic composition.

The luciferase reporter assays described here demonstrated that D6S1276 and its flanking sequences mediated considerable inter-allelic, intra-allelic and cell-specific variability on the transcriptional activity of the *BMP5 *promoter. Inter-allelic differences have been noted for a number of other intronic microsatellites [21-23]. For example, a TCAT microsatellite within intron 1 of the tyrosine hydroxylase gene was found to mediate quantitative effects on gene expression due to allelic variations in affinity for nuclear proteins with longer repeat alleles showing increased protein interaction when compared with shorter repeat alleles [21]. Thus, a possible mechanism for inter-allelic differences in the transcriptional activity of various D6S1276 alleles could be differential affinity for nuclear factors between different size D6S1276 alleles. Indeed, D6S1276 contains a consensus-binding site for proteins of the GATA family of transcription factors with the consensus WGATAR corresponding to the complementary strand of D6S1276 tAGATAGa [24].

The bone morphogenetic proteins (BMPs) constitute a subfamily within the TGF-β superfamily of secreted signaling molecules. Initially discovered as promoters of bone growth and formation, BMPs are now known to be key participants in the morphogenesis of a variety of vertebrate tissues and organs with a well-defined role in the differentiation of chondrocytes through promotion of cell proliferation and matrix synthesis [25,26]. In particular, BMP5 has been shown to play an important role in the regulation of ovarian development [27], cardiac development [28], and limb bud development [29] with a well-defined role in the differentiation of chondrocytes through promotion of cell proliferation and matrix synthesis [30,31].

In a previous study, we characterized the differential allelic expression (DAE) of the *BMP5 *gene in a variety of mesenchymal-derived tissues [32]. We discovered that not only is DAE of *BMP5 *common, but that there is also significant variability both to the existence and extent of allelic imbalance in the tissues within an individual, suggesting that *BMP5 *expression is regulated by a complex network of tissue-specific control elements. Indeed, previous work on the expression of mouse *Bmp5 *demonstrated an extensive transcriptional control network regulating the expression of mouse *Bmp5 *with a number of tissue-specific *cis*-acting regulatory sequences [33,34]. These regulatory sequences were not only shown to regulate *Bmp5 *expression in distinct skeletal structures but to also govern expression in highly discrete growth domains within these tissues [35]. Based on the results of our current study, it is plausible then that D6S1276 contributes to both the allelic imbalance and the context-specific control in human *BMP5 *expression, as we observed a number of allele-specific and cell-specific expression patterns in the mesenchymal-derived cell lines that were used in this study.

## Conclusions

The TGF-β signaling pathway has received much attention in OA genetic susceptibility recently with reports of association to functional variants in the *ASPN *gene (encoding asporin, a negative regulator of TGF-β signaling) [36-38] and the *GDF5 *gene (encoding GDF5, a member of the BMP family of signaling molecules) [39-41]. Moreover, microarray analysis of RNA extracted from bone samples of OA cases and non-OA controls demonstrated altered expression of a number of TGF-β related genes in the OA samples including *TGFB1 *(encoding TGF-β1), *BMP5*, *INHBA *(encoding activin βA, a TGF-β superfamily signaling molecule), and *FST *(encoding follistatin, a TGF-β/BMP antagonist), further strengthening the potential role of TGF-β related genes in OA susceptibility [42]. These studies in combination with our present *BMP5 *study suggest that subtle, quantitative alterations in TGF-β related signaling realized through polymorphisms affecting transcription factor binding and/or downstream interactions with binding partners is a major contributor to OA genetic susceptibility. Further interrogation of the TGF-β signaling pathway has the potential to not only reveal a large subset of genes likely to increase susceptibility to OA but also to reveal common mediators involved in transducing the signal such as transcription factors, co-activators, co-repressors, and downstream effectors that could serve as targets for therapeutic interventions.

## Competing interests

The authors declare that they have no competing interests.

## Authors' contributions

All authors were involved in drafting the article or revising it critically for important intellectual content, and all authors approved the final version to be published. Study conception and design: JMW, LS, KC, JL. Acquisition of data: JMW, LS, ZM. Analysis and interpretation of data. JMW, LS, KC, JL.

## Pre-publication history

The pre-publication history for this paper can be accessed here:

http://www.biomedcentral.com/1471-2350/10/141/prepub

## Supplementary Material

Additional file 1 **R program for BLANKET**. R program for BLANKET. This program yields a value that can be tested in Table S1 for statistical significance of the discovered shortlists. Click here for file
