# Supplementary material for: Association of a functional microsatellite within intron 1 of the BMP5 gene with susceptibility to osteoarthritis
Source: BMC Med Genet. 2009 Dec 19;10:141. doi: 10.1186/1471-2350-10-141 (PMC2807860; doi:10.1186/1471-2350-10-141)

**Supplementary Material**

**Table S1.** Primer sequences used for amplification of the *BMP5* proximal promoter sequence and associated intron 1 polymorphisms.

| **Amplified Region^a^** | **Forward Primer (5′ to 3′)** | **Reverse Primer (5′ to 3′)** |
| --- | --- | --- |
| Proximal promoter (-1783 to -472) | ttaggtaccggcgcctctaggtagagtaaag | gttgctagccagatctatgctgatctgcac |
| D6S1276 (+24415 to +24606) | gccgctagcctaggtagggatctatctatc | gccaagcttcttagacatgcctggcaaatg |
| rs921126 (+29854 to +30128) | gttgctagcctgatagtcacgtacatgg | gccaagcttcacttgccataagtag |
| rs17734678 (+44226 to +44426) | gccgctagccagatcatctgataactaac | gccaagcttcagagcaaaattcagctacgc |

**^a^** Positions are given with respect to the translation start site (GenBank accession number NM_021073).

**Table S2.** Primer sequences used for the site-directed mutagenesis of rs9475429, rs9475430, and rs9475431.

| **SNP** | **Change** | **Sense Primer (5′ to 3′)** | **Anti-sense Primer (5′ to 3′)** |
| --- | --- | --- | --- |
| rs9475429 | A to T | ctataaatacacacataaacttttctatctggtaaatatttatgttctacttgacattt | aaatgtcaagtagaacataaatatttaccagatagaaaagtttatgtgtgtatttatag |
|  | T to A | ctataaatacacacataaacttttctatcaggtaaatatttatgttctacttgacattt | aaatgtcaagtagaacataaatatttacctgatagaaaagtttatgtgtgtatttatag |
| rs9475430 | C to T | ctatctatctatctatctatctatctatctacttacctaactacctatctataaa | tttatagataggtagttaggtaagtagatagatagatagatagatagatagatag |
|  | T to C | ctatctatctatctatctatctatctacctacttacctaactacctatctataaa | tttatagataggtagttaggtaagtaggtagatagatagatagatagatagatag |
| rs9475431 | G to T | tctaatctatctatctatctatatctatctatctatctatctatctatctatctatcta | tagatagatagatagatagatagatagatagatagatatagatagatagatagattaga |
|  | T to G | tctaatctatctatctatctatatctatcgatctatctatctatctatctatctatcta | tagatagatagatagatagatagatagatcgatagatatagatagatagatagattaga |

**Table S3.** Summary of polymorphisms identified within intron 1 of *BMP5* for use in the association analysis. (A) Polymorphic microsatellites. (B) Single nucleotide polymorphisms (SNPs) and insertion/deletions (INDELS) identified from within sequences conserved between human and mouse using search criteria of at least 70% identity over at least 100 base pairs in length.

A.

| **Microsatellite** | **Chr 6 Location (bp)^a,b^** | **Repeat Unit** |
| --- | --- | --- |
| MS1 | 55793475 | TTGT |
| MS2 | 55809363 | AC |
| MS3 | 55810093 | AGAT |
| MS4 | 55814358 | TGTT |
| MS5 | 55814660 | AG |
| D6S1276 | 55823112 | TCTA |
| MS6 | 55825896 | AAATA |
| MS7 | 55826061 | TC |

B.

| **SNP/INDEL^c^** | **Chr 6 Location (bp)^a^** | **Variation** | **Conserved Region (bp)^a^** | **Sequence Identity (%)** |
| --- | --- | --- | --- | --- |
| rs17734678 | 55803335 | C/T | 55803335-55803482 | 73 |
| rs921126 | 55817700 | A/G | 55817569-55818040 | 80 |
| rs1470527 | 55819170 | T/C | 55819120-55819241 | 70 |
| rs3839430 | 55841584 | –/G | 55841501-55842105 | 78 |
| rs3798822 | 55845930 | T/G | 55845877-55845988 | 73 |
| rs3798821 | 55846211 | C/A | 55846135-55846345 | 69 |
| rs3798819 | 55846230 | C/T | 55846135-55846345 | 69 |
| rs12198673 | 55846435 | C/T | 55846404-55846572 | 71 |
| rs9382564 | 55846472 | A/C | 55846404-55846572 | 71 |
| rs3798818 | 55847012 | C/T | 55846999-55847132 | 79 |

**^a^** Sequence locations are given according to the UCSC May 2004 human reference sequence, NCBI build 35, dbSNP build 125.

**^b^** Locations given for microsatellite markers are the first nucleotide of the repeating sequence.

**^c^** dbSNP reference (http://www.ncbi.nlm.nih.gov/SNP/).

**Table S4. List of 28 conserved non-coding sequences between human and mouse located within intron 1 of human *BMP5*.**

| **Name** | **Conserved Region (bp)^a^** | **Sequence Identity (%)** |
| --- | --- | --- |
| 1 | 55792856-55792969 | 71 |
| 2 | 55795763-55795922 | 74 |
| 3 | 55800899-55801019 | 74 |
| 4 | 558023441-55802449 | 71 |
| 5 | 55802822-55803016 | 72 |
| 6 | 55803335-55803482 | 73 |
| 7 | 55808341-55808801 | 73 |
| 8 | 55817330-55817433 | 70 |
| 9 | 55817569-55818040 | 80 |
| 10 | 55819120-55819241 | 70 |
| 11 | 55820858-55821159 | 72 |
| 12 | 55822556-55822656 | 70 |
| 13 | 55826224-55826383 | 70 |
| 14 | 55834134-55834265 | 73 |
| 15 | 55834447-55834553 | 71 |
| 16 | 55838955-55839301 | 73 |
| 17 | 55839970-55840075 | 72 |
| 18 | 55841501-55842105 | 78 |
| 19 | 55842507-55842750 | 75 |
| 20 | 55843239-55843431 | 71 |
| 21 | 55844306-55844409 | 71 |
| 22 | 55844984-55845082 | 71 |
| 23 | 55845877-55845988 | 73 |
| 24 | 55846135-55846345 | 69 |
| 25 | 55846404-55846572 | 71 |
| 26 | 55846691-55846804 | 71 |
| 27 | 55846811-55846916 | 72 |
| 28 | 55846999-55847132 | 79 |

**^a^** Sequence locations are given according to the UCSC May 2004 human reference sequence, NCBI build 35, dbSNP build 125.

**Table S5.** Results of pair-wise LD statistics between SNP markers. LD statistics (*D*′ and *r*^2^) were estimated from CEPH genotyping data from the Human HapMap release 21 (http://www.hapmap.org)

| **Marker** | **LD Statistics^a^** | | | | | |
| --- | --- | --- | --- | --- | --- | --- |
|  | rs17734678 | rs921126 | rs1470527 | rs9475429 | rs9382564 | rs3798818 |
| rs17734678 |  | 1 | 1 | 1 | 0.74 | 0.74 |
| rs921126 | 1 |  | 1 | 1 | 0.58 | 0.59 |
| rs1470527 | 0.56 | 0.49 |  | 1 | 1 | 1 |
| rs9475429 | 0.59 | 0.53 | 0.96 |  | 0.70 | 0.69 |
| rs3798822 | 0.20 | 0.21 | 0.14 | 0.10 | 1 | 1 |
| rs9382564 | 0.08 | 0.04 | 0.09 | 0.04 |  | 1 |
| rs3798818 | 0.08 | 0.04 | 0.08 | 0.04 | 1 |  |

**^a^** *D′* values are shown above the diagonal, and *r^2^* values are shown below the diagonal.

**Table S6.** Allele counts and allele frequencies for haplotypes defined by the alleles of rs9475431, D6S1276, rs9475430, rs9475429, and rs1470527 in the combined case-control cohort.

| **Haplotype** | **rs9475431** | **D6S1276** | **rs9475430** | **rs9475429** | **rs1470527** | **Allele Counts (%)** | | |
| --- | --- | --- | --- | --- | --- | --- | --- | --- |
|  |  |  |  |  |  | **Combined Cohort** | | **Total Chromosomes** |
|  |  |  |  |  |  | **Cases** | **Controls** |  |
| I-A | T | (TCTA)_3_ | T | T | T | 0 (0.0) | 1 (0.1) | 1 (0.0) |
| I-B | T | (TCTA)_5_ | T | T | T | 1 (0.1) | 0 (0.0) | 1 (0.0) |
| I-C | T | (TCTA)_6_ | T | T | T | 2 (0.1) | 2 (0.1) | 4 (0.1) |
| I-D | T | (TCTA)_7_ | T | T | T | 24 (1.7) | 37 (2.4) | 61 (2.0) |
| I-E | T | (TCTA)_8_ | T | T | T | 328 (22.4) | 310 (20.2) | 638 (21.3) |
| I-F | T | (TCTA)_9_ | T | T | T | 215 (14.7) | 205 (13.3) | 420 (14.0) |
| I-G | T | (TCTA)_10_ | T | T | T | 21 (1.4) | 19 (1.2) | 40 (1.3) |
| I-H | T | (TCTA)_11_ | T | T | T | 2 (0.1) | 0 (0.0) | 2 (0.1) |
| I-I | T | (TCTA)_12_ | T | T | T | 0 (0.0) | 4 (0.2) | 4 (0.1) |
| II-D | G | (TCTA)_7_ | C | A | C | 12 (0.8) | 23 (1.5) | 35 (1.2) |
| II-E | G | (TCTA)_8_ | C | A | C | 2 (0.2) | 1 (0.1) | 3 (0.1) |
| II-F | G | (TCTA)_9_ | C | A | C | 67 (4.6) | 78 (5.1) | 145 (4.8) |
| II-G | G | (TCTA)_10_ | C | A | C | 582 (40.0) | 620 (40.3) | 1202 (40.2) |
| II-H | G | (TCTA)_11_ | C | A | C | 192 (13.2) | 213 (13.9) | 405 (13.5) |
| II-I | G | (TCTA)_12_ | C | A | C | 7 (0.5) | 20 (1.3) | 27 (1.0) |
| II-J | G | (TCTA)_13_ | C | A | C | 2 (0.1) | 3 (0.2) | 5 (0.2) |
| II-K | G | (TCTA)_15_ | C | A | C | 1 (0.1) | 0 (0.0) | 1 (0.0) |


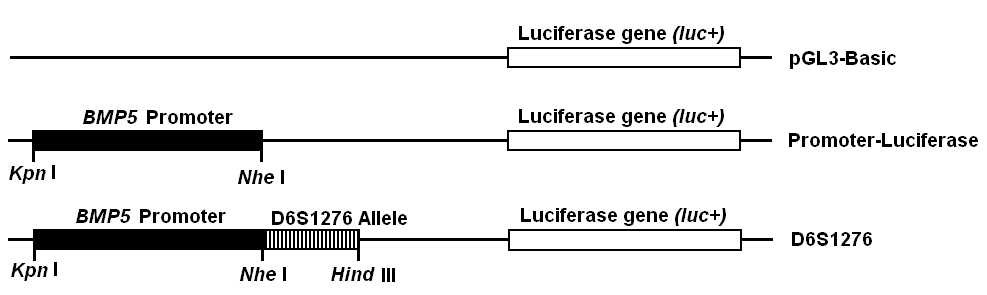
**Figure S1.** Structure of luciferase reporter vectors used in this study. These vectors were generated by cloning the *BMP5* proximal promoter into the pGL3-Basic vector to create the Promoter-Luciferase construct with subsequent subcloning of the D6S1276 alleles into this construct.


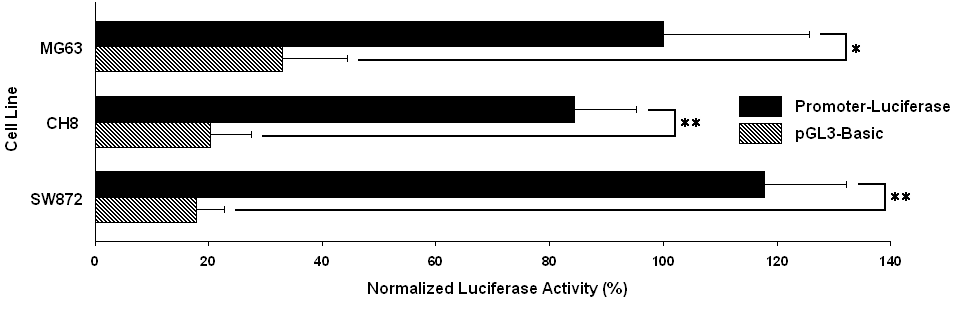
**Figure S2.** Luciferase reporter assays of the *BMP5* Promoter-Luciferase construct and the pGL3-Basic vector in MG63, CH8, and SW872 cells. The *BMP5* Promoter-Luciferase construct was generated by cloning the *BMP5* proximal promoter upstream of the luciferase gene in the pGL3-Basic vector. Transcriptional activities are given as a percentage of the activity of the *BMP5* Promoter-Luciferase construct in MG63 cells. Data shown are the mean ± SD of at least 3 experiments done in triplicate. Black bars indicate results for the *BMP5* Promoter-Luciferase construct. Checked bars indicate results for the pGL3-Basic vector. **P* < 0.0001, ** *P* < 1.0 x 10^-8^ (Student’s t-test).


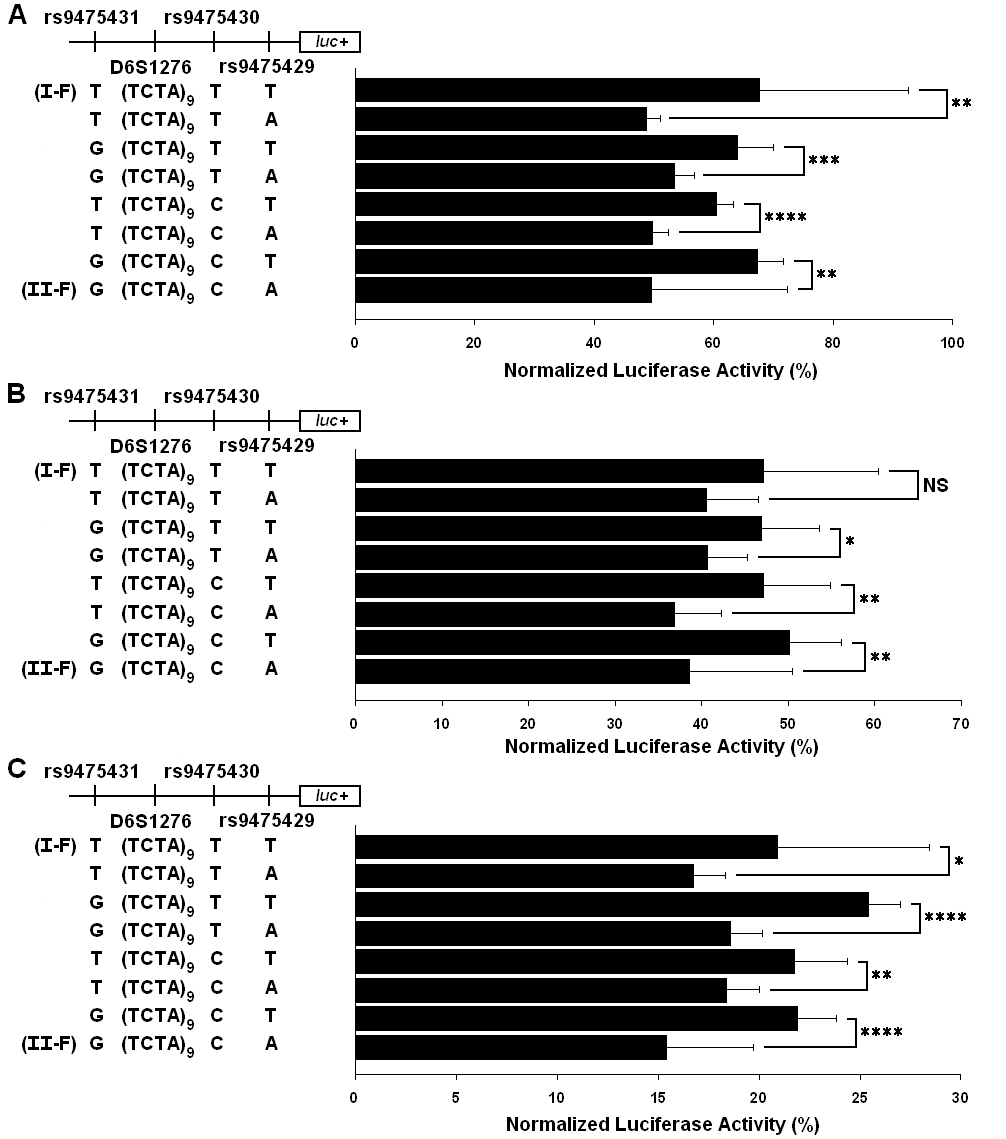
**Figure S3.** Haplotype comparison of rs9475431, rs9475430, and rs9475429 on *BMP5* promoter activity. Luciferase reporter assays were performed in MG63 (A), CH8 (B), and SW872 cells (C). Reporter constructs were generated by site-directed mutagenesis of common haplotypes I-F (TTT) and II-F (GCA). Transcriptional activities are given as a percentage of the activity of the *BMP5* Promoter-Luciferase construct for each cell line. Data shown are the mean ± SD of at least 3 experiments done in triplicate. **P* < 0.05, ** *P* < 0.01, *** *P* < 0.001, **** *P* < 2.0 x 10^-5^ (Student’s t-test). NS is not significant.

**Figure S4.** SNP comparison of rs9475431, rs9475430, and rs9475429 on *BMP5* promoter activity. Luciferase reporter assays comparing the mean transcriptional activities of haplotypes containing the T allele of rs9475431 and haplotypes containing the G allele, haplotypes containing the T allele of rs9475430 and haplotypes containing the C allele, and haplotypes containing the T allele of rs9475429 and haplotypes containing the A allele were performed in CH8 (A) and SW872 cells (B). Data shown are the mean ± SD. ** *P* = 1.1 x 10^-3^, and *** *P* = 0.020 (Student’s t-test). NS is not significant.


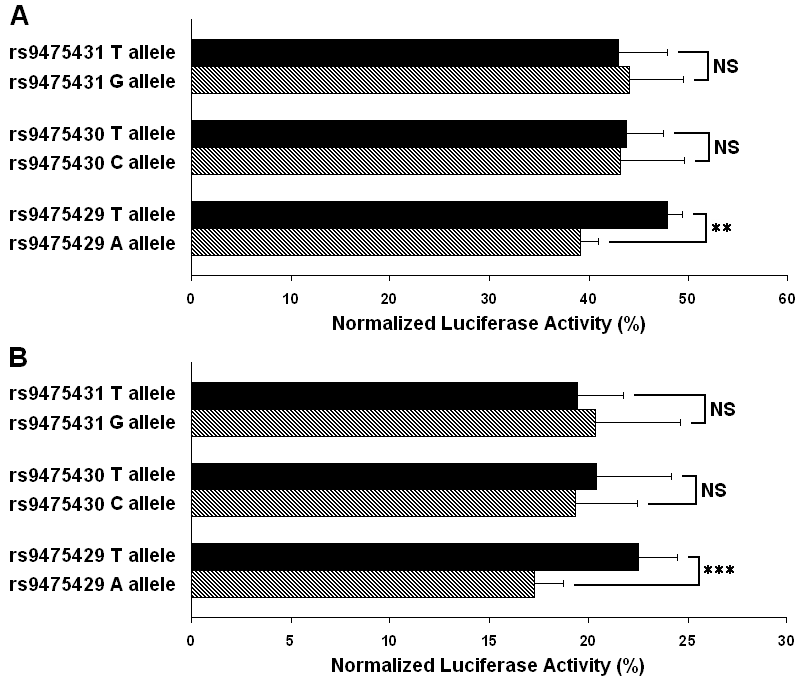

Supplement: Additional file 1 — R program for BLANKET. R program for BLANKET. This program yields a value that can be tested in Table S1 for statistical significance of the discovered shortlists. [file 1471-2350-10-141-S1.doc]
